# Regenerating a monoblock to obturate root canalsvia a mineralising strategy

**DOI:** 10.1038/s41598-018-31643-8

**Published:** 2018-09-06

**Authors:** Le Zhang, Quan-Li Li, Ying Cao, Yun Wang

**Affiliations:** 1 College & Hospital of Stomatology, Key Lab. of Oral Diseases Research of Anhui Province, Anhui Medical University, Hefei, 230032 China; 2 College of Stomatology, Wannan Medical College, Wuhu, 241002 China

## Abstract

To develop a novel strategy for sealing and obturating dental root canals by tooth-like tissue regeneration, premolars with mature root apices were freshly collected, and root canals were prepared by following the clinical protocols *in vitro*. The teeth were immersed in supersaturated calcium and phosphate solution containing gallic acid and fluoride. At certain intervals, the dental roots were taken out, and their mineral precipitates were characterised by scanning electron microscopy, energy-dispersive spectroscopy mapping, X-ray diffraction and transmission electron microscopy. The cytocompatibility of the mineralizing products were evaluated with rabbit bone-marrow-derived mesenchymal stem cells *in vitro*. Results showed that the precipitates were mainly composed of fluoridated hydroxyapatite with ahexagonal prism morphology. Fluoridated hydroxyapatite initially nucleated and grew from the root canal dentine surface to the root canal centre. The fluoridated hydroxyapatite precipitate and root canal dentine intergraded together such that the interface became hardly distinguishable. The fluoridated hydroxyapatite precipitate grew into and obturated the dentinal tubules. In the root canal, the regenerated fluoridated hydroxyapatite densely packed and bundled together with a c-axis extension. After 7 days of mineralisation, the root canal was completely obturated, and the apical foramen was sealed. The mineralizing products had good biocompatibility with the cells, and the cells grew well on the mineralized surface. Biomimetic mineralisation strategy provides a novel means to regenerate tooth-like tissue to seal the root canal system permanently other than by passive synthetic material filling.

## Introduction

Although root canal therapy (RCT) is fairly predictable and successful for pulpitis and apical periodontitis, 10-year failure rate was approximately 20%^1^. And most of these instances can be attributed to inappropriate mechanical debridement, persistence of bacteria in the canals and apex, poor obturation quality, over- and underextension of the root canal filling and coronal leakage^2^. As the final step in RCT, obturation of the root canal system may be necessarily conducted to seal the in-growth of the microorganisms originating outside the canal, entomb the residual microorganisms and their by-products and seal the fluid as nutrient for microorganisms from any source^3^. An ideal canal obturating material should adapt well to the prepared canal walls and their irregularities as a homogenous mass without dimensional changes. Obturation of the root canal system can be achieved in a number of ways, but the most commonly advocated method is the application of gutta-percha as core obturation material combined with a root canal sealer^4^. However, gutta-percha shrinks upon cooling and does not chemically adhere to the canal walls^5^. The shrinkage and dissolution of traditional sealers, such as zinc oxideeugenol or calcium hydroxide sealer, may result in a void between the dentine and canal filling materials or between the gutta-percha and sealer^6,7^. 65% of endodontic failures are caused by incomplete obturation of the root canal system^8^. In addition, the over- and underextension of root filling materials also contributes to RCT failure. Coronal leakage is also a potential factor causing endodontic failure; thus, a thoroughly sealing coronal restoration is essential. However, if no leakage ensues amongst the root filling materials and root canals, coronal leakage will not occur. Therefore, root canal obturation materials and technique need improvement.

Microleakage occurs in the interface between the dentine and sealer and between sealer and core materials. Various types of sealers have been introduced to eliminate microleakage. Examples of these sealers are glass ionomers, bioglass, polymer resins and silicone-based sealer. However, microleakage is hardly avoided during the use of glass ionomers or silicone-based sealers because of technique sensitivity attributed to the fast setting of glass ionomers and the demand of absolute desiccation in the canal, respectively^9^. Bioglass cannot directly bond to the root canal^10^. Polymer resins do not bond to and tend to pull away from gutta-percha during setting^11^. Hence, the complete sealing of the root canal system is almost impossible under the currently accepted materials and methods^12–14^. Actually, leakage cannot be prevented unless the sealer bonds to the root canal dentine and core material^15^.

With the application of dentine adhesive technology, endodontic monoblock filling technique has attracted interest in the field of endodontics. Monoblock units can be created in a root canal system by adhesive root canal sealers/resin cement or bondable coating filling materials/post^16,17^. However, the degradation of the interface between the dentine and resin adhesives make it controversial whether to improve the quality of the seal in the root fillings or to strengthen the roots^15,17^. As an apexification material, mineral trioxide aggregate (MTA) attempts to strengthen immature tooth roots and the root dentine. This material represents a contemporary version of the primary monoblock, but with only one interface between the material and the root canal dentine surface^16^. MTA does not bond to dentine, but the released calcium and hydroxyl ions of MTA interact with a phosphate-containing body fluid, resulting in the formation of apatite-like interfacial deposits. These deposits fill any gaps produced during the material shrinkage phase and enhance the frictional resistance of MTA to the root canal walls. Creating a primary monoblock within the root canal to match the elastic modulus of the root dentine has been reported to reduce the stresses that occur inside the tooth structure. As such, this strategy can be considered as an ideal method for reinforcing the root canal^17^.

In the present study, we developed a primary monoblock technique involving a mechanically homogeneous unit with root dentine through a biomimetic mineralisation strategy. We achieved a homogenous and monolithic root canal obturation without leakage between the root canal dentine by forming a thick and compact rod-like fluoridated hydroxyapatite (FHA) deposition as a monoblock that bonds tightly to the canal dentine and seals off all the community of the root canal system from the external environment.

## Results

### Characterisation of the precipitate structure and composite

**Figure 1:**
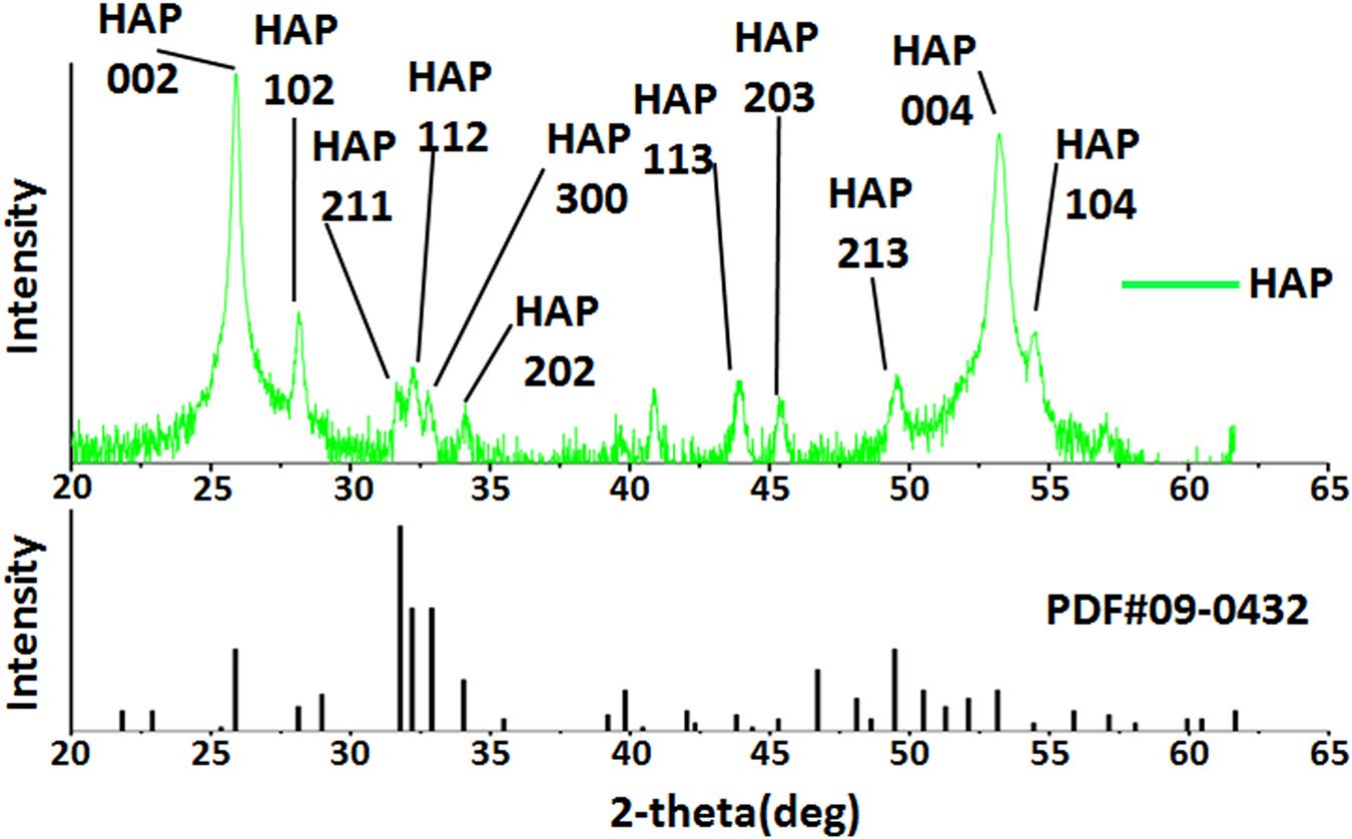
XRD spectrun of mineral precipitation on the surface of dentine slice. The peaks at 2θ = 25.879°, 28.126°, 31.773°, 32.196°, 32.902°, 34.048°, 43.804°, 45.305°, 49.468°, 53.143°, 54.440° corresponded well to the expected peaks for FHA at 002, 102, 211, 112, 300, 202, 113, 203, 213, 004 and 104 planes, respectively (JCPDS No. 09-0432). The sharp and intense 002 peak indicated that the crystals were well crystallised and oriented along their c axis.

**Figure 2:**
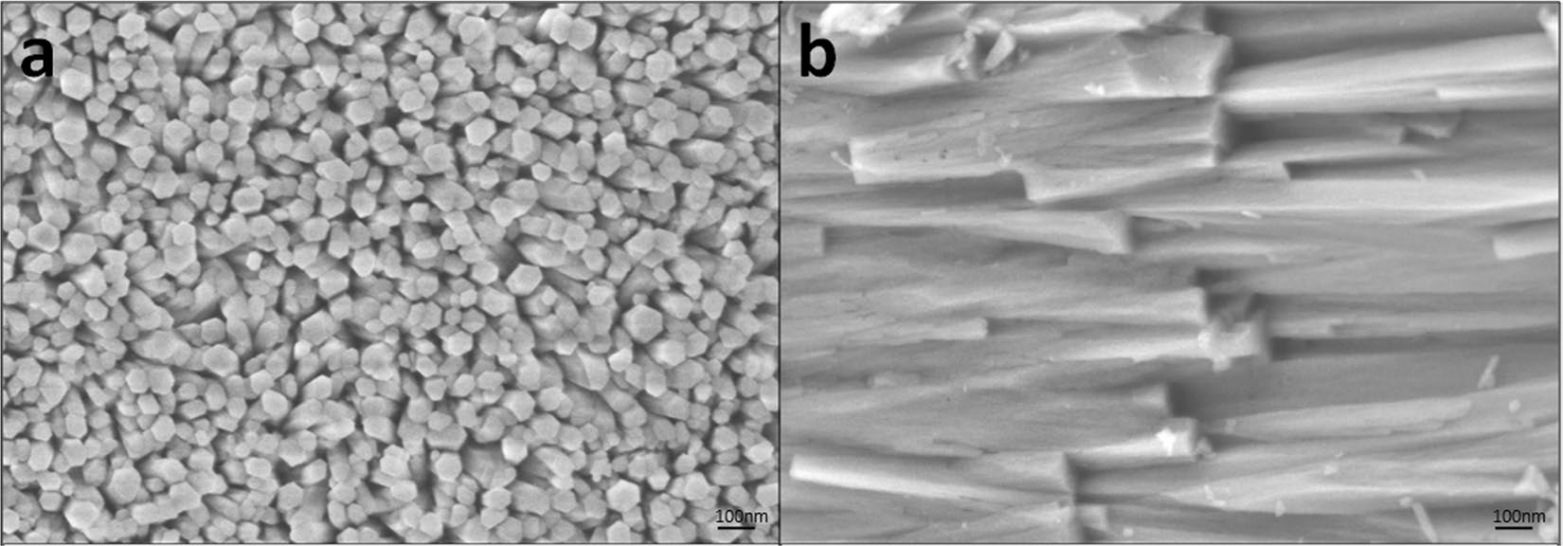
SEM micrograph of the precipitates of mineralisation for 3 days, viewed from the surface (**a**) and crosssection (**b**).

**Figure 3:**
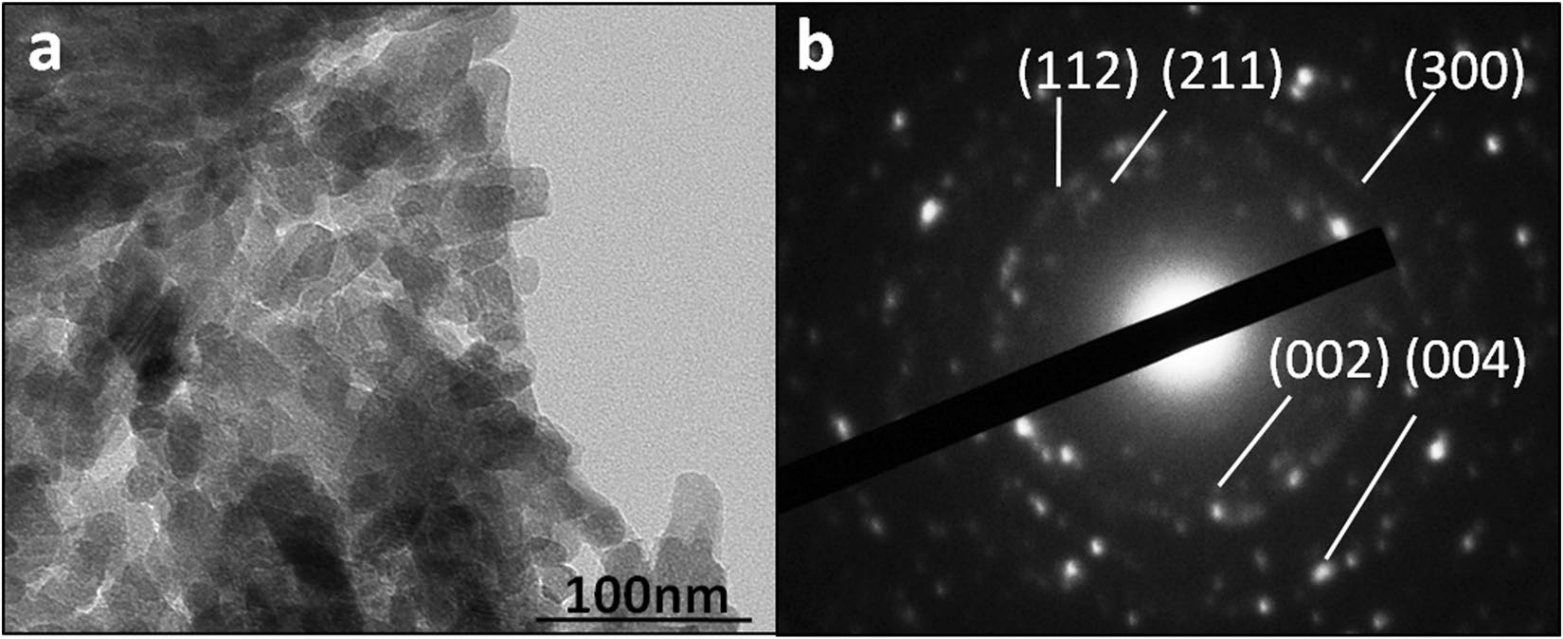
TEM micrograph (**a**) and SAED pattern (**b**) from the powder of scraped precipitates.

**Figure 4:**
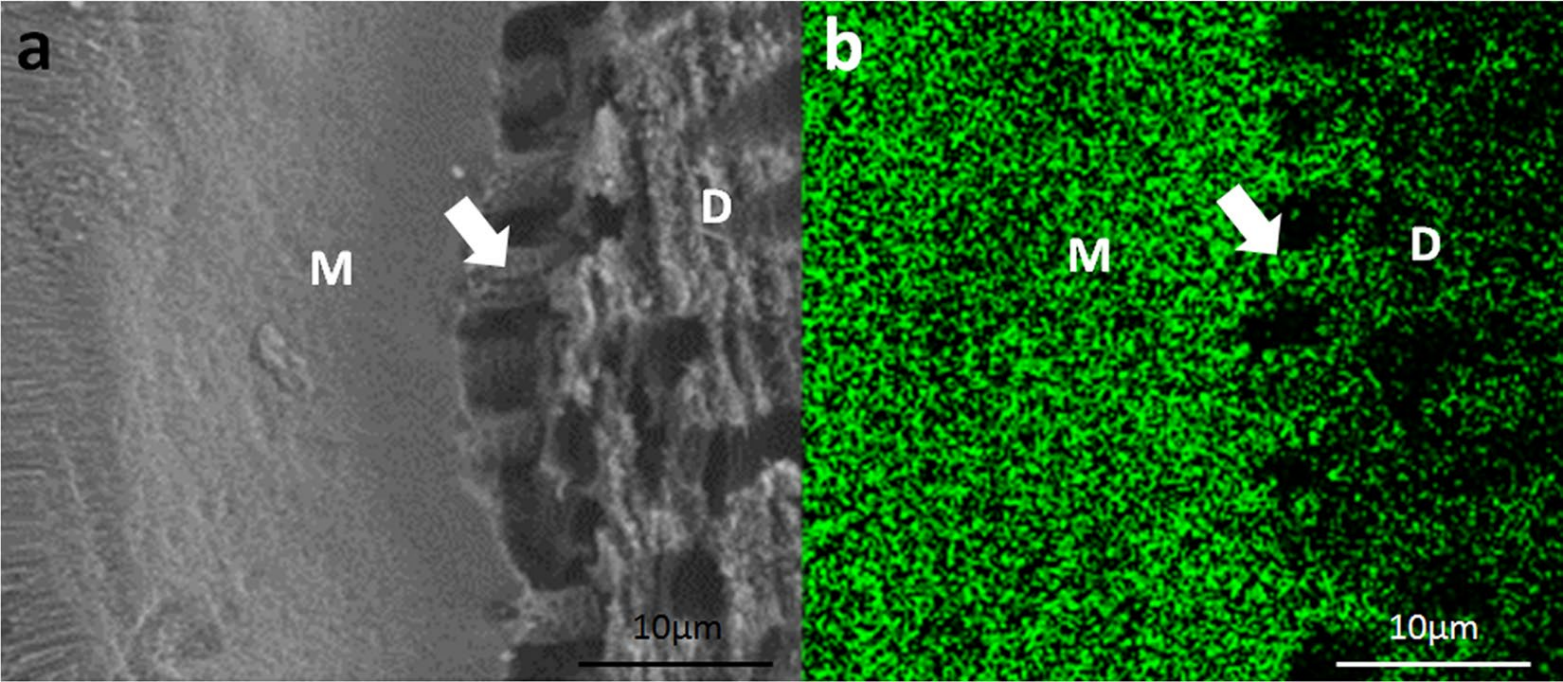
SEM and EDS-mapping microphotographs of the FHA precipitate–dentine interface after acid etching (2 days of sample mineralisation). Notes: M: mineral deposition, D: dentine, white arrow: tags. (**a**) SEM showing dentine morphology, mineral deposition and mineral tags. (**b**) EDS-mapping displaying the distribution of the fluorine element of dentine, mineral deposition and mineral tags. The concentration of green colour represents the relative amount of fluorine.

X-ray diffraction (XRD), transmission electron microscopy (TEM), scanning electron microscopy (SEM) and energy-dispersive spectroscopy mapping (EDS-mapping) for fluorine confirmed that the precipitates were mainly composed of FHA. In XRD spectrum of the precipitate grown on the dentine slice (Fig. 1), the peaks corresponded well to the expected peaks for FHA (JCPDS No. 09-0432). The sharp and intense 002 peak indicated that the crystals were well crystallised and oriented along their c axis; these results are consistent with the observations in the SEM images (Fig. 2). SAED of the TEM results from the powder of the scraped precipitates (Fig. 3) also confirmed that the rod-like crystals were FHA. SEM showed that the precipitate was composed of uniform homogenous rod-like crystals with a hexagonal prism morphology (Fig. 2) and densely packed with c-axis extension. EDS-mapping of fluorine further verified that the crystals contained fluoride (Fig. 4).

### Evaluation of the precipitate growth process and morphology

**Figure 5:**
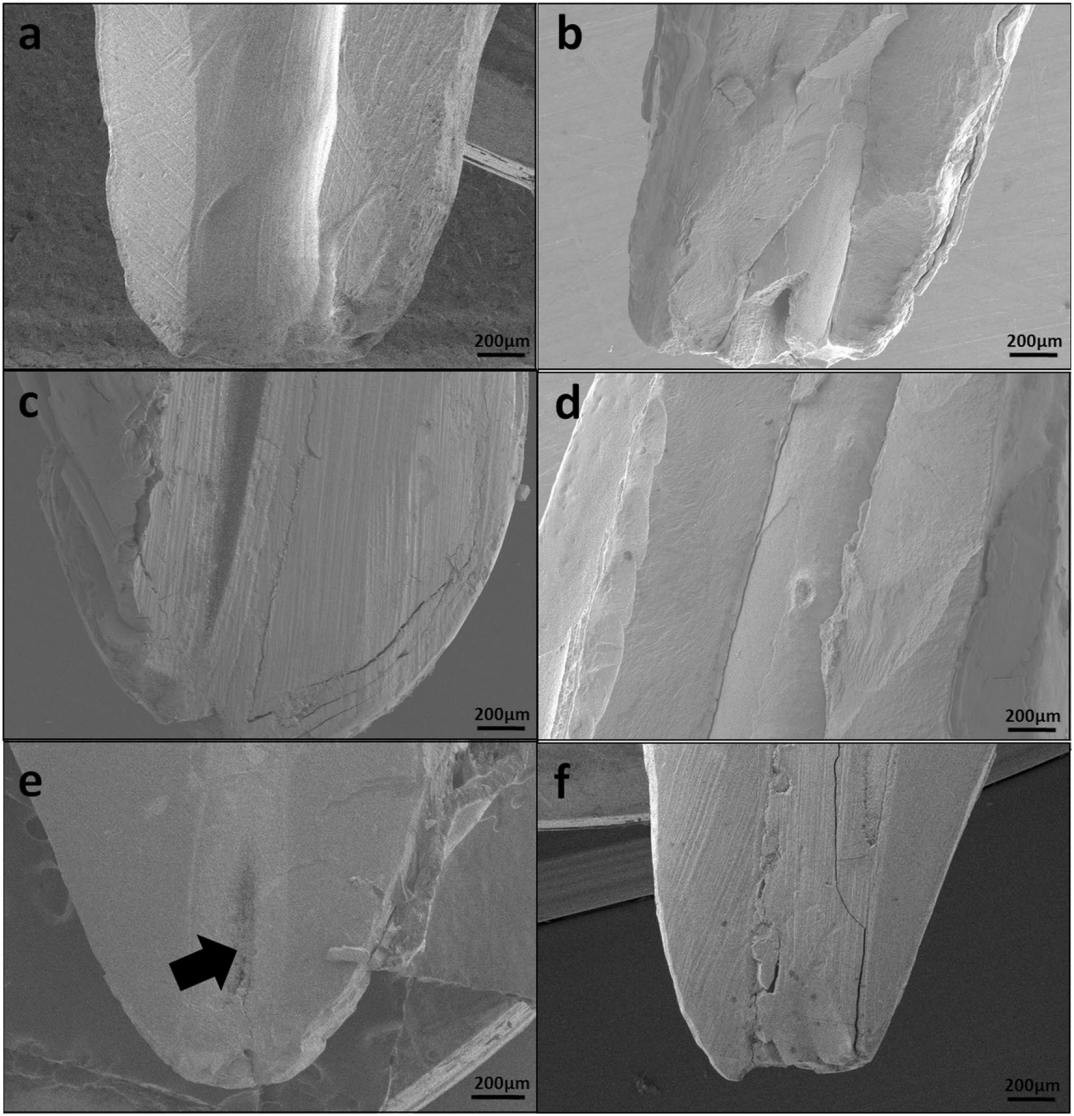
SEM shows the process of obturating the apical area of the root canal (apical foramen sealing). (**a**)Apical area of the root canal before mineralisation (sawing the root longitudinally). (**b**)The FHA precipitation growing from the root canal wall surface to the root canal centre after 3 days of mineralisation(splitting the root longitudinally). (**c**) Apical foramen was not completely obturated after 3 days of mineralisation (sawing the root longitudinally). (**d**) Longitudinally split root after 7 days of mineralisation. (**e**) Apical foramen completely obturated after 7 days of mineralisation (sawing the root longitudinally). A void remained (black arrow) at the centre of the precipitates but was isolated by mineral deposition. (**f**) Longitudinally sawed root showing the deposition obturation of the root canal after 7 days of mineralisation.

The precipitate morphology and the root canal obturation were evaluated by SEM. The FHA precipitate nucleated and grew from the dentine surface to the centre of the root canal and finally obturated the entire root canal (Fig. 5). After 7 days of mineralisation, the root canal was completely obturated, and the apical foramen was sealed (Figs 4e and 5d,f). Although voids remained in the centre of the precipitates in some samples, they were surrounded by mineral deposition (Fig. 5e). No seam or gap was visible between the root canal and the precipitate. A monolithic monoblock was formed to obturate the canals.

**Figure 6:**
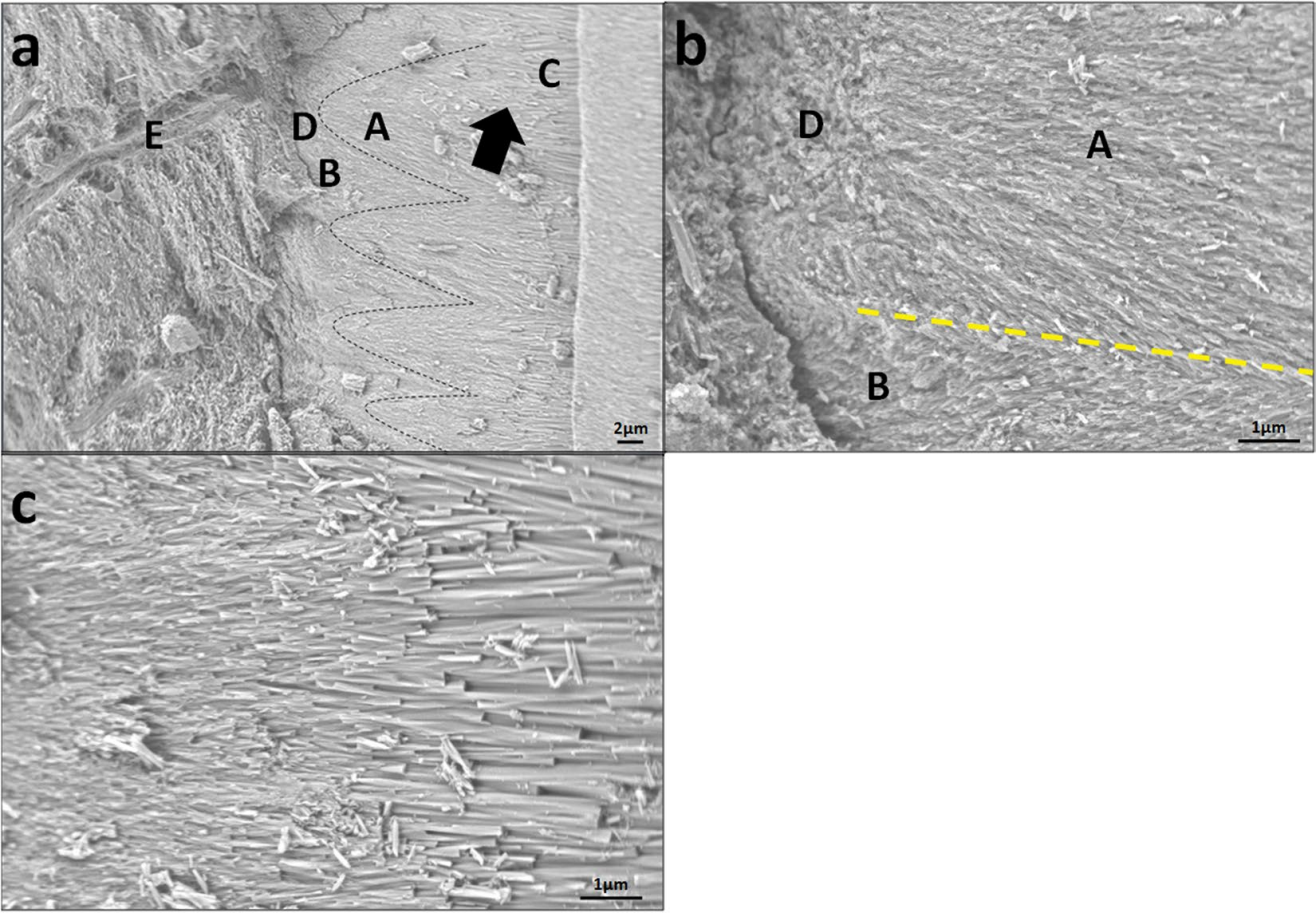
SEM micrographs of the transverse section of the interface between the layer of precipitates and the root canal wall and the crystal morphology changes from the root canal wall surface to the root canal centre. Notes: A: mineral precipitate on the intertubular dentine surface, B: mineral deposition in the dentinal tubule, C: enlargedcrystals with atypical hexagonal prism morphology in parallel orientations, D: intertubular dentine, E: dentinal tubule, black dotted line: ‘U type’ structure on the canal dentine surface, black arrow: the size of the crystals tended to increase, orange arrow: mineral tags in the dentinal tubule. (**a**) Interface between the precipitate layer and the root canal wall. (**b**) Magnification of (**a**) showing the interface and the morphology of the precipitate near the interface. (**c**) Crystal morphology changes from the root canal wall surface to the root canal centre.

The precipitates and dentine surface of the root canal intergraded together such that the interface became hardly distinguishable and showed a tight junction (Fig. 6). The mineral precipitate thoroughly matched with the canal dentine. Near the interface, the deposition showed an array of ‘U type’ morphology (indicated by the black dotted line in Fig. 6, region A). This result was achieved probably because the *in situ* growth of the FHA crystals on the intertubular dentine surface (Fig. 6, region D) was separated by dentinal tubules at the start to form an isolated radial ‘island’ morphology until the lateral crystal growth came in contact and fused with the adjacent radical ‘island’. Meanwhile, crystal growth expected to originate from the dentinal tubule (Fig. 6, region B) was suppressed by the radical ‘island’ when they grew to reach the radical ‘island’. The interface between the deposition regions of a and b (Fig. 6, yellow dotted line) can be clearly distinguished by the different crystal growth directions. Thus, the desynchrony of crystal growth on the canal dentine surface and in the dentinal tubule may also lead to the ‘U type’ structure.

**Figure 7:**
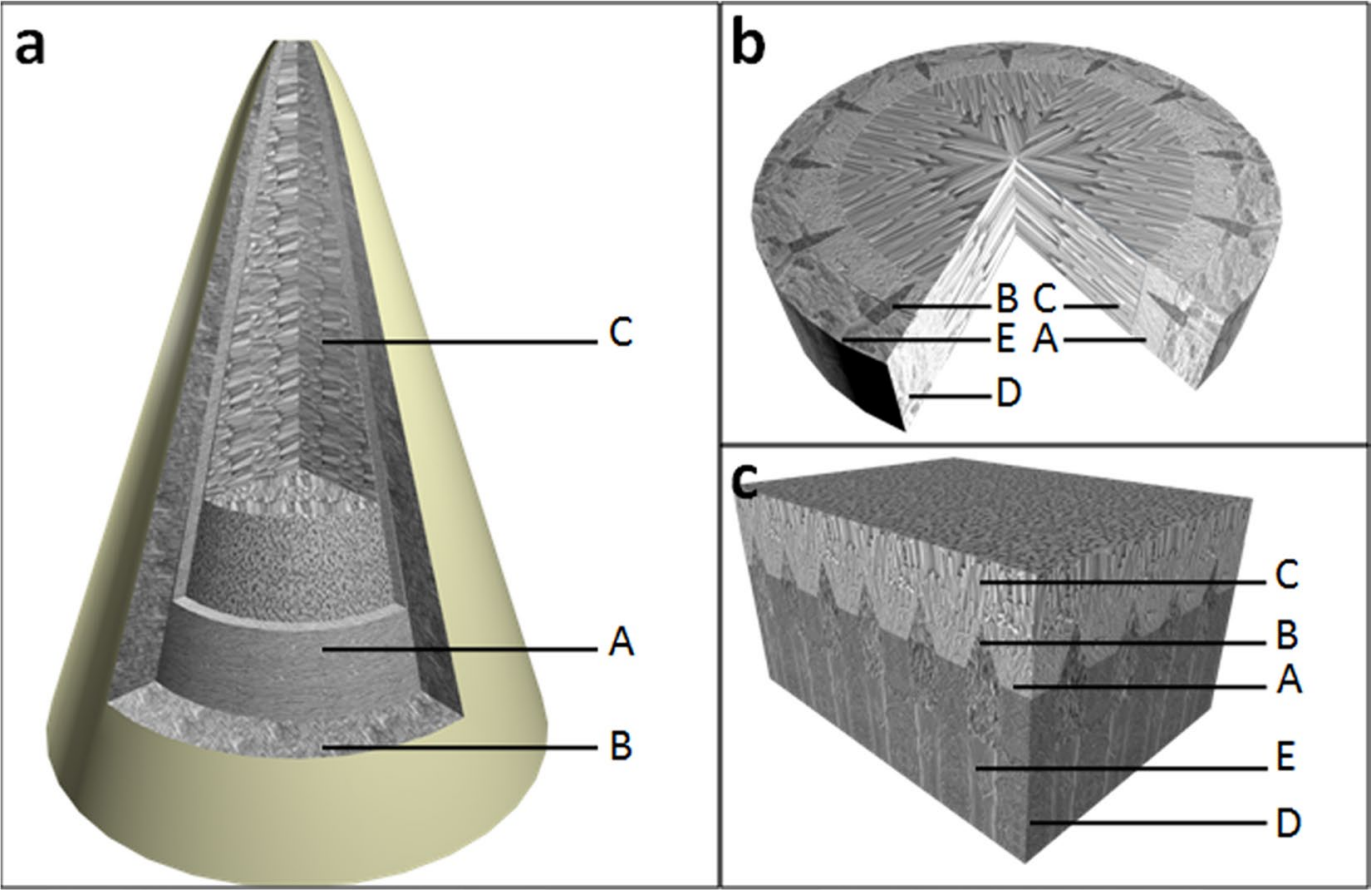
Schematic of the FHA crystal growth process. Notes: A: mineral precipitate on the intertubular dentine surface, B: mineral deposition in the dentinal tubule, C: enlargedcrystals with atypical hexagonal prism morphology in parallel orientations, D: intertubular dentine, E: dentinal tubule. (**a**) showing the precipitate in root canal. (**b**) cross-section of (**a**). (**c**) magnification of (**b**) showing the ‘U type’ structure on dentin surface.

The crystal size tended to enlarge from the areas of the dentine surface of the root canal to the root canal centre (Fig. 6c). The initial growth of the crystal clusters was limited by a radical ‘island’ and the anisotropic root dentine structure until the ‘island’ fused. As a result, the crystals were arranged radically and vertically near the anisotropic dentine surface to maintain a small size. Afterwards, all the crystals grew in parallel orientations and extended along the c-axis to transform into enlarged crystals with a typical hexagonal prism morphology. Figure 7 summarizes schematically crystal growth process of FHA in root canal.

To stereoscopically observe the interface structure of the mineral precipitate and dentine, the splitting specimens were etched with phosphoric acid and scanned by SEM and EDS-mapping (Fig. 4). Dentine demineralisation was noted, and the precipitates gained a higher resistance to acid etching than the dentine. At the precipitate–dentine interface, a mass of parallel tags appeared (Fig. 4a). It is suggested that the precipitates grew into and obturated the dentinal tubules. EDS-mapping for fluorine further verified that the FHA precipitates grew into the dentine tubules by penetrating the mineral solution, which allowed the growth of the FHA crystals in dentinal tubules (Fig. 4b). The high fluorine concentration was distributed uniformly throughout the mineral precipitate regardless of the location (within the root canal or dentinal tubules). The fluorine concentration in the dentine was much lower than that in the precipitate.

### Cytocompatibility of the precipitates

**Figure 8:**
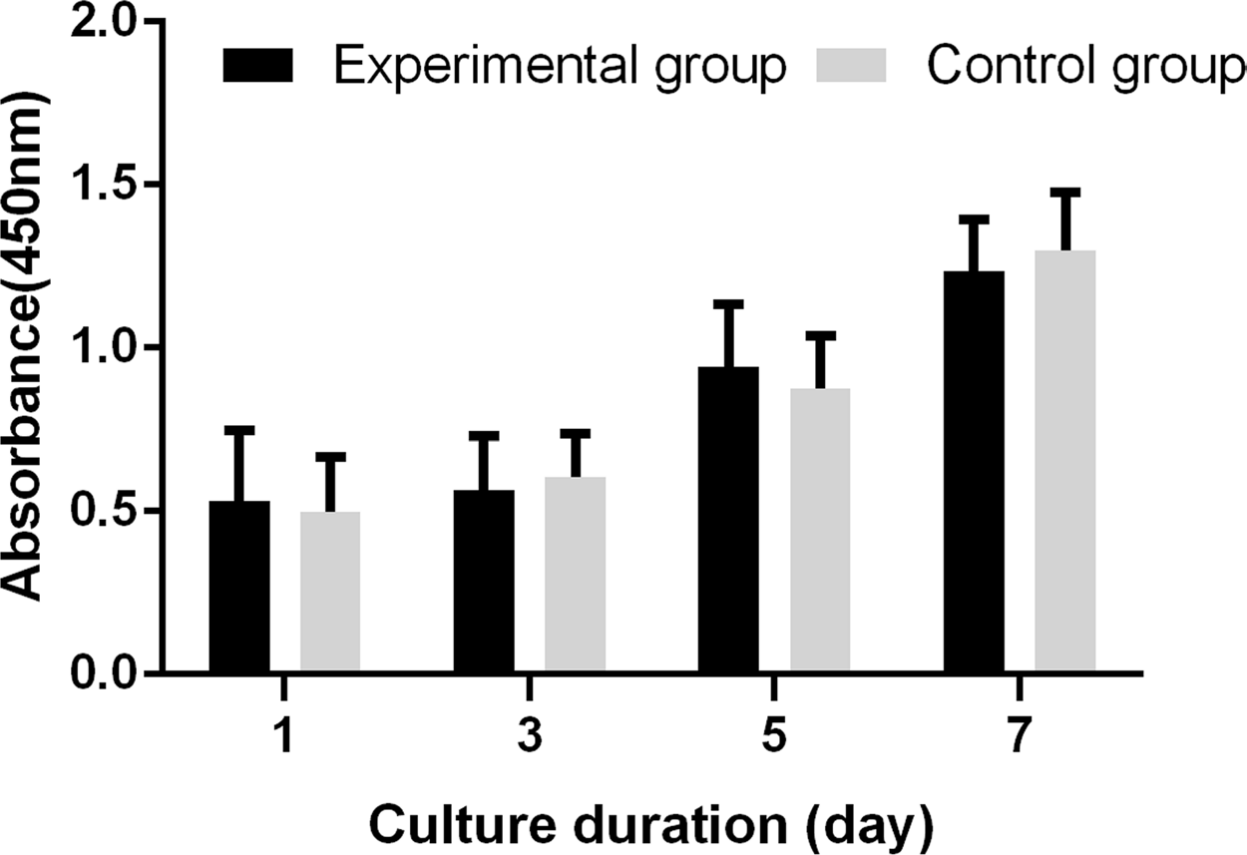
Cell proliferation measured by CCK-8. No significant difference in absorbance were found between experimental group and control group during the cell culturing span from day 1 to day 7 (P > 0.05)

**Figure 9:**
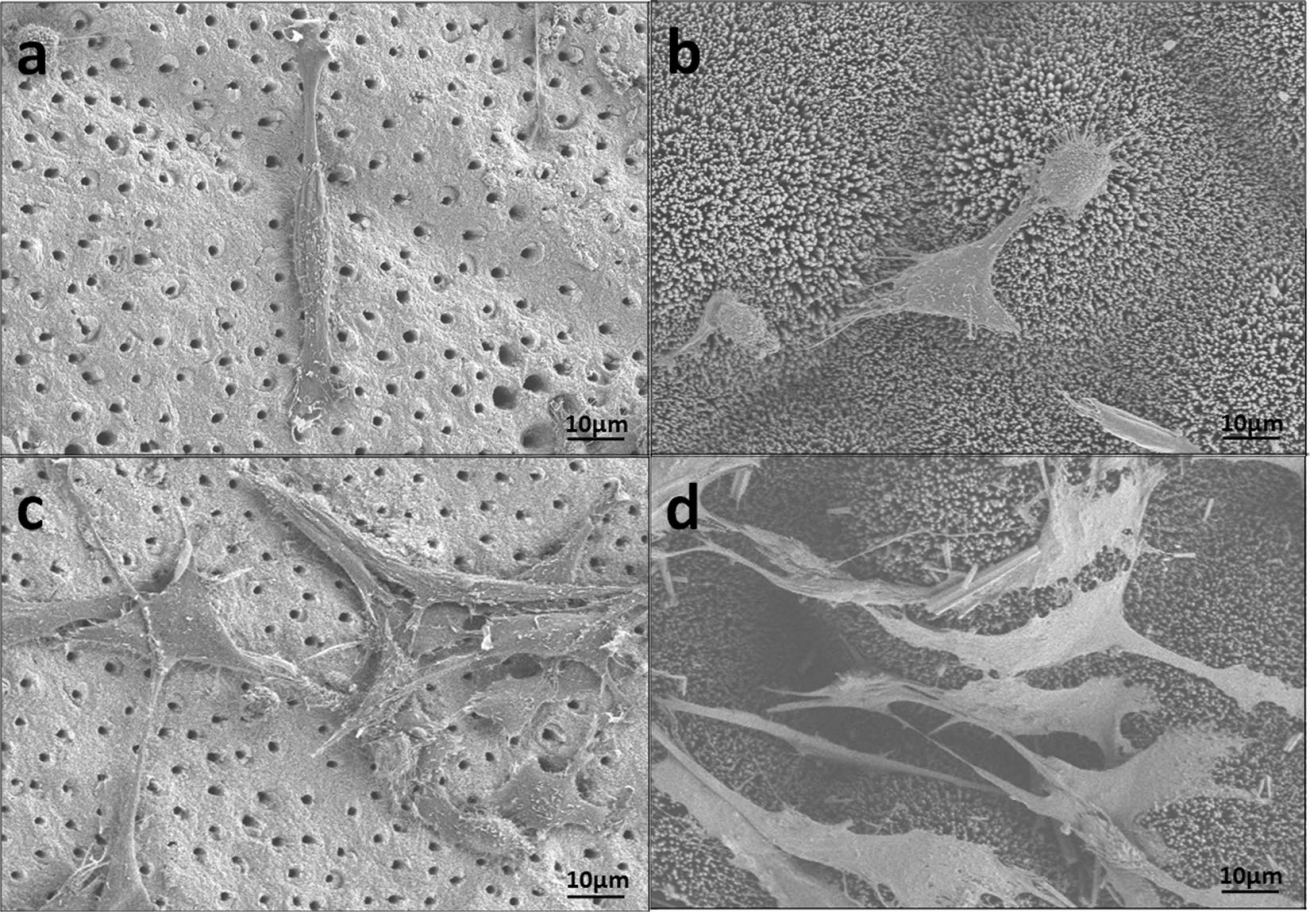
SEM of cells on dentin and HA surface and (**a**,**c**) Spindle-shaped rabbit-BMSCs attached to dentin slice with filopodia at 1-day incubation and 3-day incubation respectively. (**b**,**d**) Well spread rabbit-BMSCs with obvious cytoplasmic extension attached to HA precipitates with filopodia and lamellipodia at 1-day incubation and 3-day incubation respectively.

The leach solution of the precipitate was collected to evaluate the cytocompatibility of the precipitates. In experimental group rabbit bone-marrow-derived mesenchymal stem cells (rabbit-BMSCs) were cultured in leach solution, while in control group cells were culture in culture medium. Cell proliferation at 1, 3, 5 and 7 days was measured by Cell Counting Kit-8 (CCK-8) assay. There was no significant difference between experimental group and control group during the cell culturing span (P > 0.05) (Fig. 8). SEM images showed that the rabbit-BMSCs well adhered to the precipitated surface and dentin surface. After 1 day culture, the spindle-shaped cells well adapted to substrate surface and attached to each other. After 3 days culture, the cells grew and proliferated with more filopodia and lamellipodia extending. Compared with cells seeded on dentin surface, the cells on precipitated surface obviously spread with more cytoplasmic extension (Fig. 9).

## Discussion

Currently, the dental root canal is only passively obturated by synthetic material filling in RCT clinics. Even with all the advances in materials and filling techniques, poor obturation quality, over- and underextension of the root canal filling and leakage between the materials and root canal dentine surface or within the filling materials are often the main causes of failure in RCT clinics. Although the monoblock technique has been proposed for its advantages^16^, this method remains as a passive filling technique for obturation and cannot completely seal the root canal system permanently. With the currently accepted materials and methods, permanent and complete sealing of the root canal system cannot be achieved.

Regenerating enamel-like and dentin-like microstructures through a molecular biomimetic approach is highly interesting in present clinical dentistry. We have provided some promising methods for regenerating tooth-like tissue for treating tooth defects via a self-healing mechanism^18–23^. Herein, we present a novel concept of regenerating tooth-like tissue to seal the root canal system permanently other than passive synthetic material filling by using a mineralising strategy. In this method, the following aspects are addressed.

Firstly, contaminants of the root canal dentine surface are removed, and the dentine surface is activated. During the root canal treatment, the canal surface is covered with organic and inorganic substances, odontoblast fragments, microorganisms and necrotic debris known as the smear layer^24–26^. Complete seamless integration, strong chemical binding and micromechanical interlocks between mineral precipitate and dentine can be achieved by removing the smear layer. Meanwhile, mineralisation on the dentine surface can be promoted by eliminating the organics on the dentine surface and smear because the inorganic hydroxyapatite substrate is much more active and induces hydroxyapatite formation. We treated the dentine surface with 17% EDTA and 5.25% NaOCl. In brief, EDTA chelation causes demineralisation of the smear layer and root dentine surface and exposes collagen fibres and other non-collagen proteins simultaneously^24,25,27^. The residual organic tissue of the smear layer and the exposed collagen fibres and non-collagen proteins of the root dentine were also dissolved by NaOCl etching^28–30^. Only apatite remained on the surfaces of the canal dentine and dentinal tubule. The residual crystal in the dentine surface can act as a seed mineral crystal to facilitate FHA crystal nucleation and epitaxial growth. This process leads to a thickened layer of mineral deposition and finally obturation of the canal system.

Secondly, a key issue of biomimetic mineralisation is using an organic molecule to control mineral crystal growth. Herein, we used gallic acid to promote biological mineralisation. Studies have found that the catecholamine moieties of the pyrogallol groups can anchor to the tooth surface bound to calcium ions, enriching the interface with calcium ions and therefore facilitating hydroxyapatite growth^31^. Meanwhile, gallic acid also exerts an antimicrobial effect on pathogenic bacteria^32^ and is highly interesting for root canal obturation.

Thirdly, regenerating tooth-like tissue to obturate the root canal prevents reinfection caused by the leakage of tooth filling materials. The regenerated FHA crystals were initiated from the canal dentine surface and integrated with the dentine mineral crystals (Fig. 6a). No leakage existed at the dentine–precipitate interface. By contrast, mineralisation grew into the dentinal tubules. These tags contributed to the sealing of the dentinal tubule, improving the binding property of the precipitates to the dentine and the mechanical retention of the mineral deposition.

Finally, the precipitate is mainly composed of FHA crystals. The densely packed and bundled crystals formed an enamel-like prism structure. Therefore, they may have a similar expansion coefficient with enamel. This strategy for obturating the root canal by regeneration of tooth-like tissue may prevent the defects caused by conventional root canal filling, such as the over- and underextension of the root canal filling and leakage.

The monoblocks created in the root canal spaces are classified as primary, secondary or tertiary depending on the number of interfaces present between the bonding substrate and bulk core material^16^. On the basis of this classification, a primary monoblock involves only one interface that extends circumferentially between the material and the root canal wall. This monoblock is considered as the ideal type. MTA represents the primary monoblock, butdoes not bond to dentine. Our strategy may be applied to primary monoblocks. The regenerated tooth-like tissue may reinforce the root canal and may be viewed as an ideal obturation. However, this work is only a primary study; the mineralising method must still be developed. For instance, a hydrogel mineralising agent maybe used to replace the solution agent^19,23^ or the mineralisation speed may be accelerated with the aid of a direct electric field^22,23^. The biomimetic mineralization still requires a comprehensive research program for its clinic application.

Through biomimetic mineralisation strategy, densely packed and bundled FHA crystals formed enamel-like prism structures in the root canal. The regenerated tooth-like tissues integrated with the root dentine and grew into dentinal tubules, and have good biocompatibility. Our results provide a novel concept for regenerating tooth-like tissue to seal root canal systems permanently other than using passive synthetic material filling.

## Methods

### Teeth collection

Premolars with mature root apices were freshly collected after obtaining informed consent approved by patients in the Stomatological Hospital of Anhui Medical University. The teeth with root fractures, root canal calcification or existing caries were excluded. The remaining 20 teeth were then preserved in 1% sodium hypochlorite for 1 day and then carefully cleaned by an ultrasonic scaler and stored in normal saline solution at 4 °C prior to root canal preparation.

### Preparation of root canals and dentin slices

Teeth were decoronated at the cemento-enamel junction with a diamond saw (SYI-160 MTI) under slow speed with a constant water supply. Radicular pulp tissue was then removed with a barbed broach (MANI, Japan). Canals were inserted with a 15 K-file until the file tip is visible at the apical foramen. The canal lengths were then determined by the distance from the reference point to the file tip. The working length was determined and recorded by subtracting 1.0 mm from the canal length. The canals were shaped and cleaned with a hand protaper nickel titanium file. The coronal two-thirds of the canals were prepared from Sx to S2, and the apical third was prepared from F1 to F3. After each use of the file, the canals were irrigated copiously with normal saline. Once the preparation was finished, the teeth were treated with 17% ethylenediaminetetraacetic acid (EDTA) solution for 1 min, followed by 5.25% NaOCl for 5minto remove the smear layer on the canal dentine surface. The teeth were rinsed with deionised water in the presence of ultrasound three times, after the EDTA and NaOCl treatments.

Twenty dentin slices from the remaining dental crowns were prepared and polished with 1000-grit silicon carbide paper under running water. The slices were treated with 17% ethylenediaminetetraacetic acid (EDTA) solution for 1 min, and 5.25% NaOCl for 5 min, followed by deionised water in the presence of ultrasound.

### Mineralisation of root canals and dentin slices

The freshly etched and cleaned canals and fourteen dentin slices were placed in a 500 mL beaker containing 400 mL calcification solution (5.83 × 10^−3^ M CaCl_2_, 3.5 × 10^−3^ M K_2_HPO_4_, 1.17 × 10^−3^M NaF, 135.7 × 10^−3^ M NaCl, 5.5 × 10^−3^ M gallic acid, buffered by Tris 90.9 × 10^−3^ M, pH 6.4) under constant magnetic stirring at 37 °C. The calcification solution was refreshed daily. After the mineralisation interval, the specimens were removed, rinsed and subjected to ultrasound with deionised water.

The precipitates on the surface of seven dentin slices were scraped and pulverised. One piece of mineralized dentin slice was used for XRD. The remaining six mineralized dentin slices, six dentin slices without mineralisation, and the scraped precipitate power were sterilized by high-pressure steam sterilizing device for further cytocompatibility test using.

### Characterisation of the mineralisation precipitates

#### Scanning electron microscopy (SEM) and energy-dispersive spectroscopy (EDS)

To expose the precipitates, the samples were prudently prepared in different ways as follows. To maintain apical foramen integrity for the evaluation of apical obturation, one specimen after 3 days of mineralisation and two specimens after 7 days of mineralisation were precisely sawed into two halves longitudinally with a diamond saw and cleared. SEM was utilized to evaluate root canal obturation by the precipitates. To maintain the original morphology of the precipitates and the interface between the precipitate and root canal dentine, three specimen after 3 days of mineralisation and one specimens after 7 days of mineralisation were split longitudinally into two halves with a chisel. SEM was utilised to observe the microstructural morphology of mineral precipitates, root canal obturation by the precipitates and the interface junction between the root canal structure and mineral precipitates. To examine the substructure of the interface, one specimen was etched with 8.5% phosphoric acid for 7 s after being splitted. EDS-mapping was utilised to evaluate the distribution of fluorine elements in the precipitates and root dentine. For SEM observation, all the above-mentioned specimens were rinsed with deionised water, dehydrated with graded ethanol and subjected to critical point drying (Quorum K850,England). The specimens were then coated with a thin layer (3 nm thickness) of pure gold by using an ion sputtering unit of 22 mA for 30 s in a vacuum apparatus (SCD 050, Germany) and examined by SEM and EDS-mapping (Gemini 500, Germany) operating at 3 kV.

#### Transmission electron microscopy (TEM)

To characterise the crystal structure of the precipitates, the precipitates were scraped from the root canal surface without reaching the dentine. The precipitates were then pulverised, dispersed in alcohol and observed by TEM (Jeol-2010 Japan) at 200 kV. The morphology and selected-area electron diffraction (SAED) of mineral deposition were obtained.

#### X-ray diffraction (XRD)

XRD was further used to determine the crystal structure of the precipitates. To satisfy the demand for a sufficient scanning area for XRD analysis, a dentine slice was acquired, and the surface was treated with EDTA and NaOCl. The dentine slice was mineralised similar to that performed on the root canal dentine surface above. The mineral precipitate was analysed by XRD (Rigaku TTR-III) under Cu–Kα radiation (λCu–Kα = 0.1541841 nm, radiation at 40 kV and 200 mA) over the 2θ range of 20° to 80° (time per step: 0.15 s and step size: 0.02°/s). The obtained experimental patterns were compared with the documented XRD patterns of FHA (PDF#09-0432).

### Cytocompatibility of the precipitates

#### Isolationand culture of rabbit-BMSCs

One male New Zealand rabbit(age: 90–120 days; body weight: 2.1 kg; the Department of Anhui Experimental Centre of Anhui Medical University) was used in this study, and all procedure were conducted in compliance with the National Institutes of health guide for the care and use of laboratory animals and approved by Experimental Animal Ethics Committee of Anhui Medical University. Firstly, the rabbit was euthanized with an overdose of 5% chloral hydrate. DMEM (HyClone) supplemented with 10% fetal bovine serum (Thermo Fisher Scientific) and 1% penicillin/streptomycin (Thermo Fisher Scientific) was used to flush out marrow in separated bilateral femurs and tibias after the bone were cut off at epiphysis, and then the isolated cells were cultured at 37 °C in a 5% CO_2_ incubator. Four days later, the nonadherent cells were rinsed away with PBS three times, and then fresh DMEM with 1% fetal bovine serum and 1% penicillin/streptomycin was added. The cells were passaged with 0.25% trypsin/EDTA after reaching 80–90% confluency. Besides, the culture medium was refreshed every 2 days, and cells of third passages were used for further study.

#### Cell proliferation activity

Cell proliferation activity was evaluated with CCK-8 (Dojindo Laboratories Inc., Kumamoto, Japan) assay^33^. The pulverized precipitate was incubated in DMEM with 1% fetal bovine serum and 1% penicillin/streptomycin at 37 °C for one day. The concentration of the precipitate in DMEM is 25 mg/ml. Then leach solution was obtained by filtration of the mixture with Sterile Syringe Filters. The rabbit-BMSCs were seeded in four 96-well plate at a density of 4 × 10^4^/ml and incubated in leach solution for experimental group and culture medium for control group respectively for 1, 3, 5 and 7 days. At the selected incubation periods, the samples were washed with PBS and then incubated in 100 μl DMEM supplemented with 10 μl CCK-8 solution for 3 h. Then, the absorbance was measured using MQX200 absorbance microplate reader (Bio.Tek, USA) at 450 nm (n = 9 in each group). The optical density (OD) absorbance value was analyzed by GraphPad Prism 6 and compared by t-test statistically.

#### Cell morphology observation on the precipitates

Six mineralized dentin slices as experimental group and six dentin slices as control group (prepared as above) were placed in 24-well plate, and cultured DMEM medium with 1% fetal bovine serum, 1% penicillin/streptomycin, and cell density of 4.0 × 10^4^/ml at 37 °C. After one and three days of incubation, the samples were gently rinsed with PBS three times to remove unattached cells, and then fixed with 2.5% glutaraldehyde in PBS for 24 hour at 4 °C. Subsequently, the dentin slices were washed with PBS again, followed by graded ethanol and critical point dying. The cell morphology on the surface was observed by SEM.

### Ethical approval and informed consent

#### Ethical approval

The research protocol was approved by the local Independent Ethics Committee. The Ethics Committee of Anhui Medical Univerity: No 20150251.

#### Accordance

The methods were carried out in accordance with the relevant guidelines and regulations.

#### Informed consent

Informed consent was obtained from all individual participants included in the study.

## Data Availability

The datasets generated during and analysed during the current study are available from the corresponding author on reasonable request.
